# Supplementary material for: Distribution of Human T-Lymphotropic Virus (HTLV) and Hepatitis C Co-infection in Bahia, Brazil
Source: PLoS One. 2020 Jul 21;15(7):e0223087. doi: 10.1371/journal.pone.0223087 (PMC7373273; doi:10.1371/journal.pone.0223087)
Supplement: Attachment — Submitted filename: Response_to_reviewers.docx [file pone.0223087.s003.docx]

PONE-D-19-25685R2
Distribution of Human T-Lymphotropic Virus (HTLV) and Hepatitis C Co-infection in Bahia, Brazil.
PLOS ONE

Dear Editor,

Thank you very much for analyzing our manuscript. The replies to Editor’s comments are below:

 Reviewer #2: The authors have mostly addressed my concerns and critiques in the revised submission, and I commend their attention to these. I have the following minor issues that I think need addressing to make the manuscript publishable.

**Question 1 - Reviewer #2:** The authors need to be explicit in the Methods section that this study used data from anti-HCV test results indicating exposure to HCV and that they are using this as a proxy for infection in their co-infection estimates. While they correctly noted in the limitations that clearance would result in fewer infections that estimated, this could be more explicitly stated in methods. Contrary to what they note, anti-HCV is not used globally to indicate infection, it is used to indicate exposure and previous infection. Many current systematic reviews use or estimate infection based on RNA results, or impute it based on expected clearance rates. Also as more people are treated, we can expect anti-HCV to be an even larger over-estimate of infection.

**Reply**: We have included the following sentence in the methods section:

Before: no information.

After (line 127-130): The prevalence of HTLV / HCV coinfection was determined by taking into account the presence of HTLV and HCV antibodies. The presence of anti-HTLV indicates infection, while the presence of anti-HCV might indicate exposure and / or infection.

**Question 2 - Reviewer #2:** Lines 127-128: age) . authors say that they 'adjusted' the estimates. Adjusted for what? if its not adjusted, for example for age, then just say the rate per 100,000 and remove the "adjusted".

**Reply:** We thank the reviewer by his/her observation. We have removed “adjusted”. The rate means the number of infected individual per 100.000 habitants.

Before: “The ~~adjusted~~ rate of coinfection was expressed as the number of individuals infected per 100,000 inhabitants.”

After (line 130-131): “The rate of coinfection was expressed as the number of individuals infected per 100,000 inhabitants.”

**Question 3 - Reviewer #2:** The final conclusion is still weak: how can this data inform prevention? Just hoping it will is not enough - what are ways that this paper can be used beyond their descriptive contribution?

**Reply**: We have modified the conclusion as follows:

Before: “In conclusion, the present study found that at least 14% of the individuals infected with HTLV in Bahia also harbor HCV. Coinfection was concentrated in males who resided in the microregions of Salvador, Ilhéus-Itabuna and Porto Seguro, all hotspots for HTLV infection, although HTLV and HCV are widespread in the state. It is our hope that these findings will provide support for the implementation of measures to prevent infections caused by these viruses, especially in areas where higher rates of HTLV/HCV were described.”

After (line 276-287): “In conclusion, the present study evaluated a large number of individuals in Bahia, considered one of the states in Brazil most affected by HTLV infection. We found that at least 14% of the individuals infected with HTLV also harbor HCV. Coinfection was concentrated in males who resided in the microregions of Salvador, Ilhéus-Itabuna and Porto Seguro, all considered hotspots for HTLV infection, despite HTLV and HCV being widespread throughout the state. It is our hope that these findings will provide support for the implementation of preventive measures against the spread of these viruses, especially in areas where higher rates of HTLV/HCV coinfection were described. Moreover, as the presence of HTLV can negatively impact the course of HCV infection, the surveillance of active cases should be a priority in order to provide early treatment. The identification, control and prevention of the main risk factors associated with HTLV/HCV coinfection can lead to efficacious actions in a variety of epidemiological contexts specific to each affected region.”
